# P-1238. AUC You Later: Comparison of Bayesian and First-Order Estimations of Vancomycin Area Under the Curve in Pediatrics

**DOI:** 10.1093/ofid/ofaf695.1430

**Published:** 2026-01-11

**Authors:** Joo Eun Jun, David S Burgess, Hope Brandon, Katie B Olney

**Affiliations:** University of Kentucky College of Pharmacy, Lexington, Kentucky; University of Kentucky, Lexington, KY; University of Kentucky HealthCare, Lexington, Kentucky; University of Kentucky HealthCare, Lexington, Kentucky

## Abstract

**Background:**

Consensus guidelines recommend therapeutic dose monitoring of vancomycin in pediatric patients to target a 24-hour area under the curve (AUC_24_) of 400-600 mg*hr/L. Recommended methods for estimation of AUC_24_ include the use of Bayesian software, with one or two concentrations, or first-order calculations using two concentrations. Limited data exist to compare these methods, particularly in pediatric populations. This study aimed to compare calculated AUC_24_ using first-order equations with two drug concentrations at steady state to Bayesian two- and one-concentration estimations.

**Figure:**
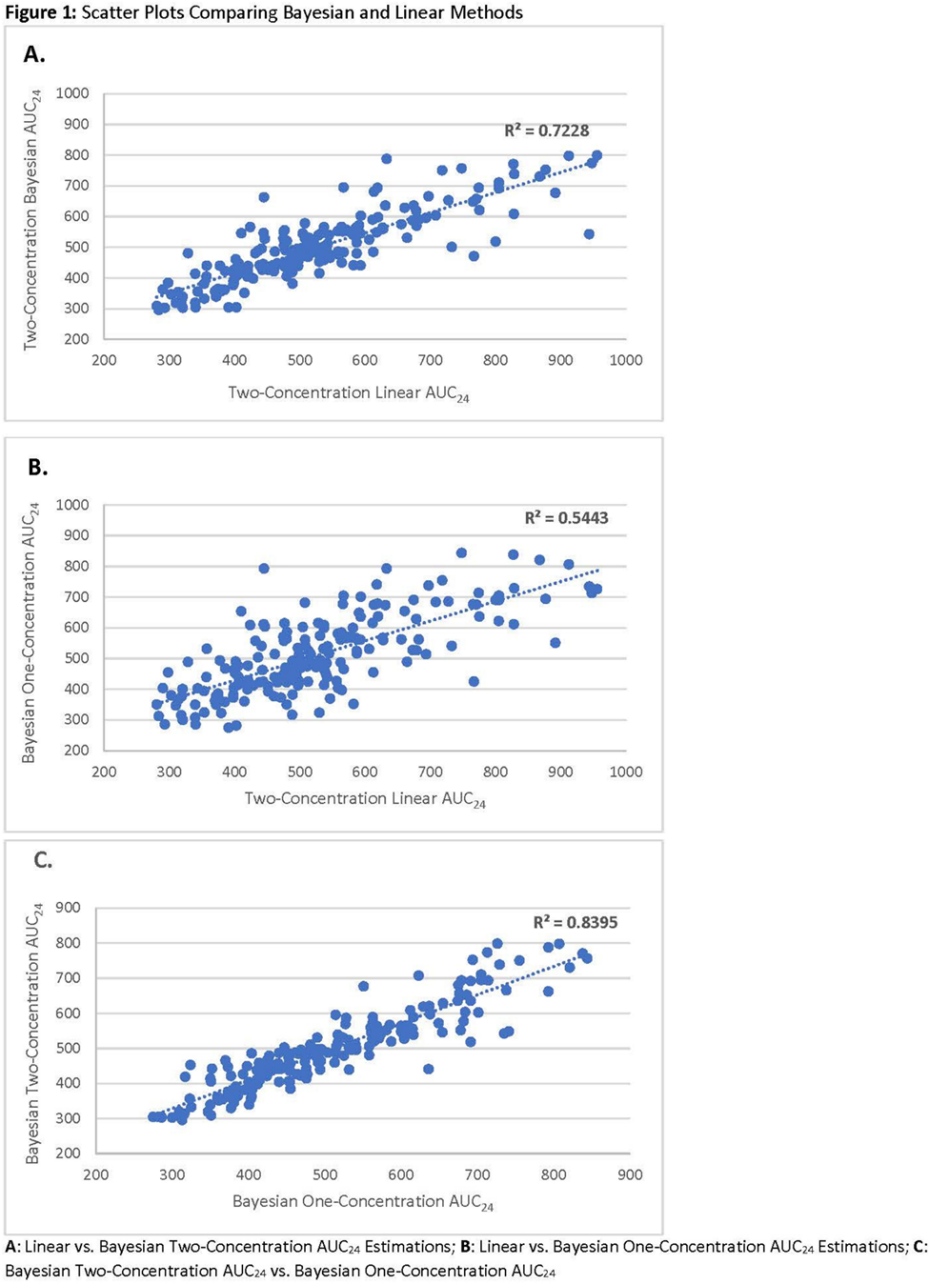

**Figure 2:**
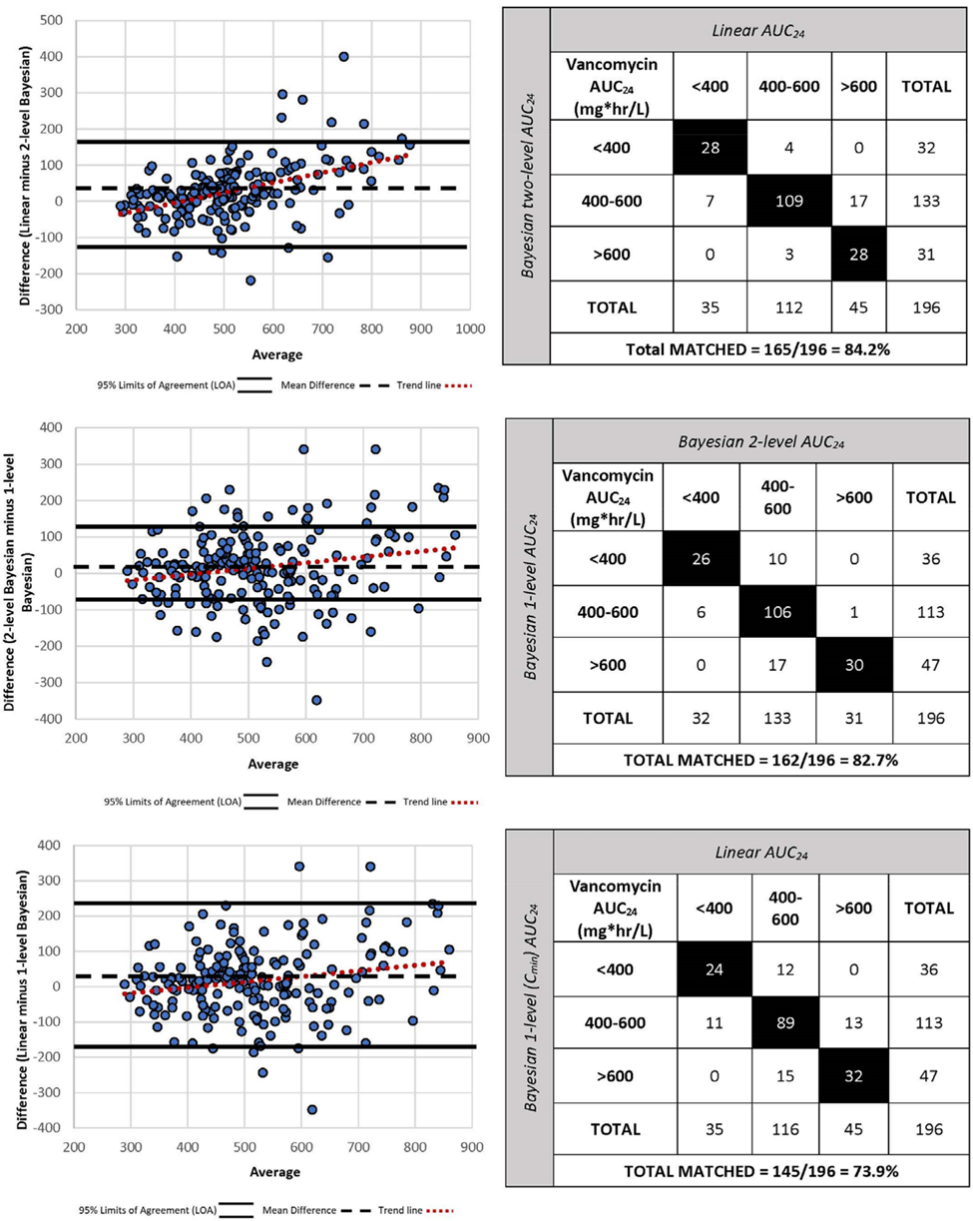
Bland Altman Plots and Categorical Agreement

**Methods:**

This was a retrospective review of hospitalized pediatric patients (< 18 years) receiving intravenous vancomycin from 2020-2022. Patients were screened if they had two concentrations collected at steady-state within 96 hours of initiation. Patients with baseline renal dysfunction were excluded. Pharmacokinetic parameters and AUC_24_ were estimated using InsightRx^TM^ Bayesian software incorporating the Colin model and first-order equations. Pearson’s correlation and clinical agreement (based on AUC_24_ classification) were used to compare methods (Figure 1). Bland-Altman plots were used to assess mean difference (MD) and 95% limits of agreement (LOA) (Figure 2).

**Results:**

Overall, 196 patients (22 neonates, 17 infants, 114 children, and 42 adolescents) were included in the final analysis. Agreement was observed between linear and Bayesian two-concentration methods (84.2%; R^2^=0.723) and Bayesian two-concentration and one-concentration methods (82.7%, R^2^=0.839). Some variability was noted with 95% LOA -123 to 178 (MD=27 mg*hr/L) and -88 to 110 (MD=11 mg*hr/L), for the respective comparisons. Lower agreement was noted between linear and Bayesian one-concentration methods (73.9%, R^2^=0.544), and demonstrated the greatest amount of variability with 95% LOA -179 to 211 (MD=16 mg*hr/L).

**Conclusion:**

Linear and Bayesian two-concentration methods demonstrated reasonable agreement with acceptable variability and may be considered comparable to estimate AUC_24_. Similarly, Bayesian two-concentration and one-concentration methods demonstrated reasonable agreement with acceptable variability, supporting the comparability of these methods to estimate AUC_24_.

**Disclosures:**

All Authors: No reported disclosures

